# MNDA, a PYHIN factor involved in transcriptional regulation and apoptosis control in leukocytes

**DOI:** 10.3389/fimmu.2024.1395035

**Published:** 2024-04-12

**Authors:** Stefania Bottardi, Taylorjade Layne, Ailyn C. Ramòn, Norreen Quansah, Hugo Wurtele, El Bachir Affar, Eric Milot

**Affiliations:** ^1^ Maisonneuve-Rosemont Hospital Research Centre, University of Montreal, Centre Intégré Universitaire de Santé et de Services Sociaux (CIUSSS) de l’Est-de-l’Île de Montreal, Montreal, QC, Canada; ^2^ Department of Medicine, Université de Montréal, Montréal, QC, Canada

**Keywords:** MNDA, PYHIN factors, innate immunity, transcription control, apoptosis, genotoxic stress

## Abstract

Inflammation control is critical during the innate immune response. Such response is triggered by the detection of molecules originating from pathogens or damaged host cells by pattern-recognition receptors (PRRs). PRRs subsequently initiate intra-cellular signalling through different pathways, resulting in i) the production of inflammatory cytokines, including type I interferon (IFN), and ii) the initiation of a cascade of events that promote both immediate host responses as well as adaptive immune responses. All human PYRIN and HIN-200 domains (PYHIN) protein family members were initially proposed to be PRRs, although this view has been challenged by reports that revealed their impact on other cellular mechanisms. Of relevance here, the human PYHIN factor myeloid nuclear differentiation antigen (MNDA) has recently been shown to directly control the transcription of genes encoding factors that regulate programmed cell death and inflammation. While MNDA is mainly found in the nucleus of leukocytes of both myeloid (neutrophils and monocytes) and lymphoid (B-cell) origin, its subcellular localization has been shown to be modulated in response to genotoxic agents that induce apoptosis and by bacterial constituents, mediators of inflammation. Prior studies have noted the importance of MNDA as a marker for certain forms of lymphoma, and as a clinical prognostic factor for hematopoietic diseases characterized by defective regulation of apoptosis. Abnormal expression of MNDA has also been associated with altered levels of cytokines and other inflammatory mediators. Refining our comprehension of the regulatory mechanisms governing the expression of MNDA and other PYHIN proteins, as well as enhancing our definition of their molecular functions, could significantly influence the management and treatment strategies of numerous human diseases. Here, we review the current state of knowledge regarding PYHIN proteins and their role in innate and adaptive immune responses. Emphasis will be placed on the regulation, function, and relevance of MNDA expression in the control of gene transcription and RNA stability during cell death and inflammation.

## The PYHIN protein family and inflammation

Inflammation is a protective response produced by immune and non-immune cells in response to injury or infection, to restore tissue homeostasis through various repair processes (1). Inflammation can either be acute or chronic. Chronic inflammation, which is also referred to as long-term inflammation, is a disorganized form of inflammation sustained in time. It is common to various diseases, including cancers (reviewed in (2)). Acute inflammation is the rapid innate immune response to pathogen infection or tissue damage. Acute inflammation consists of two phases, initiation, and resolution. Initiation is characterized by the massive recruitment of neutrophils to the site of infection or tissue damage. Once the insult has been eliminated, neutrophils undergo cell death, and their recruitment diminishes. Monocytes involved in neutrophil clearance then accumulate at the site of infection or injured tissue. Both the initiation and resolution of acute inflammation are tightly regulated, and disruption of either phase of acute inflammation can have severe consequences (3, 4).

Type I and type II interferons (IFN) are critical for both phases of acute inflammation and for chronic inflammation. Their signalling leads to strong expression of IFN-stimulated genes (ISGs), which encode proteins that play a key role in innate and adaptive immune responses to pathogen infection (5, 6), and are also called upon during sterile inflammation induced in response to sterile cell death or tissue damage. IFN production and signalling are therefore strictly regulated to adequately respond to these insults (7). MNDA is a member of a large family of IFN inducible proteins, namely, the IFN inducible p200/hematopoietic IFN-inducible nuclear protein with a 200-amino-acid repeat (Ifi-200/HIN-200) domain (8–10). MNDA was first identified as a 55 kDa non-histone basic nuclear protein in the acute promyelocytic leukaemia cell line HL-60 (11, 12), and was subsequently found to be highly expressed in the human myeloid lineage, including primary myeloblasts, promyelocytes, peripheral monocytes, granulocytes and macrophages (13, 14). It is also expressed in several lymphoblastoid cell lines as well as in primary human leukaemia and lymphoma cells (14–18). MNDA expression has been shown to be strongly upregulated by type I IFN in myeloid and lymphoid cells (19).

**Figure 1:**
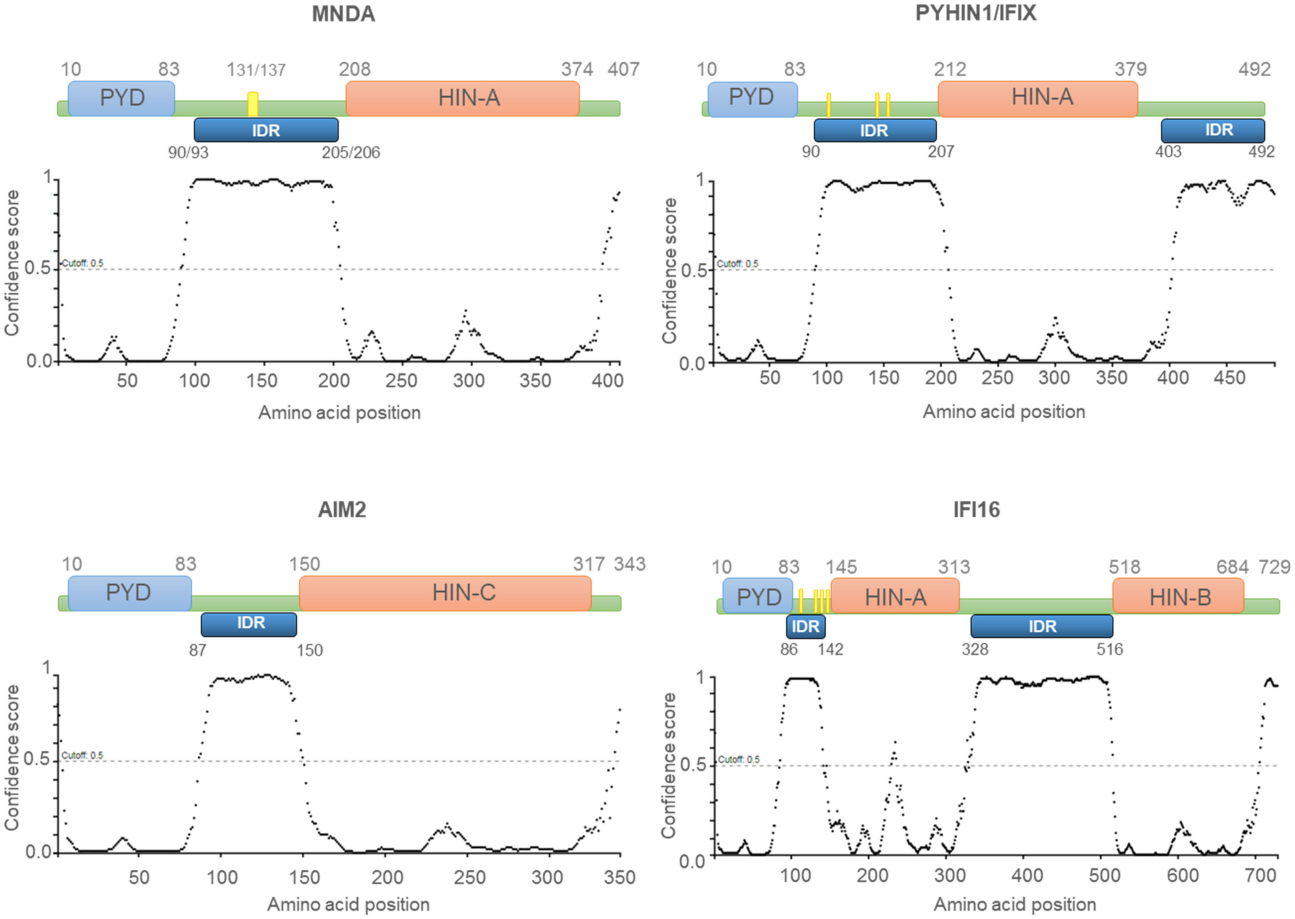
Human PYHIN proteins. Schematic diagram of the PYHIN protein structure with amino acid position. PYHIN proteins have a conserved domain architecture comprised of one PYRIN domain (PYD; light blue boxes), one or two hematopoietic IFN-inducible nuclear protein with a 200-amino-acid repeat (HIN-200; light orange boxes) domain/s, and at least one intrinsically disordered region (IDR; dark blue boxes). MNDA IDR consists of a stretch of 115/113 amino acids, spanning from residue 90/93 to residue 205/206. Except for AIM2, which has a predominant cytoplasmic distribution, the other PYHIN members contain either a multipartite nuclear localization signal (NLS) (PYHIN1/IFIX and IFI16) or a putative NLS (MNDA) (yellow boxes). IDR prediction was performed with PSIPRED (http://bioinf.cs.ucl.ac.uk/; 01/2024) and the results are plotted as scatter graphs. The canonical sequences of the PYHIN factors and the identification of their conserved domains were retrieved from the national library of medicine (NCBI) (https://www.ncbi.nlm.nih.gov/) and UniProt (https://www.uniprot.org/) databases. Reference sequences are as follows: MNDA: myeloid cell nuclear differentiation antigen [Homo sapiens]; NCBI Reference Sequence: NP_002423.1; UniProt ID: P41218. AIM2: Interferon-inducible protein AIM2 isoform 1 [Homo sapiens]; NCBI Reference Sequence: NP_004824.1; UniProt ID: O14862. IFI16: gamma-interferon-inducible protein 16 isoform 1 [Homo sapiens]; NCBI Reference Sequence: NP_001193496.1; UniProt ID: Q16666. PYHIN1/IFIX: Pyrin and HIN domain-containing protein 1 isoform alpha 1 [Homo sapiens]; NCBI Reference Sequence: NP_689714.2; UniProt ID: Q6K0P9. X-axis: amino acid number and position; Y-axis: confidence score.

Most Ifi-200/HIN-200 family factors are reported to act as pattern-recognition receptors (PRRs), which are critical for the sensing and binding of invading pathogen DNA or cytosolic self-DNA (20). The Ifi-200/HIN-200 protein family includes a sub-group of highly homologous murine [Ifi202a, Ifi202b, Ifi203, Ifi204, Ifi205, and Ifi206 (8)] and human (10, 14, 19, 21, 22) proteins. The latter includes IFN-γ inducible protein 16 (IFI16), absent in melanoma 2 (AIM2), IFN-inducible protein X/pyrin and HIN domain-containing protein 1 (IFIX/PYHIN1), and MNDA. In addition to the C-terminal HIN-200 domain(s), these proteins are characterised by an N-terminal PYRIN domain/domain in apoptosis and interferon response (PYD/DAPIN) (23, 24) (**Figure 1**). The organization of the PYD and HIN-200 domains is similar among these proteins, which are collectively referred to as the AIM2-like receptors (ALRs) or the PYHIN (contraction of PYRIN and HIN-200 domains) family (25).

Human PYHIN factors play important roles in the: (i) regulation of cell survival; (ii) innate and adaptive immune responses; and (iii) host resistance to tumours and viral infections (26–29). Some PYHIN factors were demonstrated to exert a critical function for exogenous DNA sensing, which can lead to the induction of IFNs and ISGs, or inflammasome regulation (30, 31). Sustained activation of IFN-mediated cellular responses, in particular those associated with inflammation-induced cell death, are drivers of autoimmune disorders such as systemic lupus erythematosus, systemic sclerosis, and Sjögren’s syndrome (32, 33). The mechanisms controlling IFN production and ISG induction are therefore of paramount importance to regulate tissue homeostasis, innate and adaptive immune responses, and to sustain immune surveillance of tumours.

## Structural characteristics of human PYHIN factors

The protein domains PYD and HIN-200 are critical for the biological functions of PYHIN factors. The PYD is a conserved motif composed of 80 amino acids, which can promote protein homo- and hetero-oligomerization (20, 24, 34). This domain is found in several proteins involved in signalling pathways associated with apoptosis, cell cycle and inflammation [reviewed in (8, 27, 29)]. Indeed, in response to sensing pathogen-associated molecular patterns (PAMPs) and damage-associated molecular patterns (DAMPs), cytoplasmic PRRs containing PYDs can assemble to form a multimolecular platform known as the inflammasome (35). In turn, this leads to the activation of proinflammatory caspases and triggers the inflammatory cascade, which is essential to the innate immune response [reviewed in (36)].

The HIN-200 domain is highly conserved among mammals (37). Based on sequence similarities, three distinct classes of HIN-200 domains, HIN-A, HIN-B and HIN-C, have been described (**Figure 1**). The HIN-200 domains contain a tandem pair of β barrels or oligonucleotide/oligosaccharide binding (OB) formed by 70-80 amino acid residues (37–39), and is involved in protein interactions (39, 40). HIN-200 domains can also bind nucleic acids including single-stranded (ss) and double-stranded (ds) DNA via electrostatic interactions (20, 38, 41) between positively charged amino acids of the domain and phosphate groups of the DNA backbone (20). Despite their sequence similarity, the capacity of individual HIN-200 domain to bind specific DNA molecules is variable. Fluorescence polarization assays demonstrated that the interaction between the HIN-200 domain and the DNA is influenced by ionic strength but not by the DNA sequence (20). The affinity of these domains for nucleic acids can also be modulated by intra-molecular conformation of the protein. For instance, biomolecular interaction studies using alpha screen assays have established that while the HIN-B domain of IFI16 is sufficient for binding dsDNA, the presence of both HIN-A and HIN-B domains strengthen this association (42).

**Figure 2:**
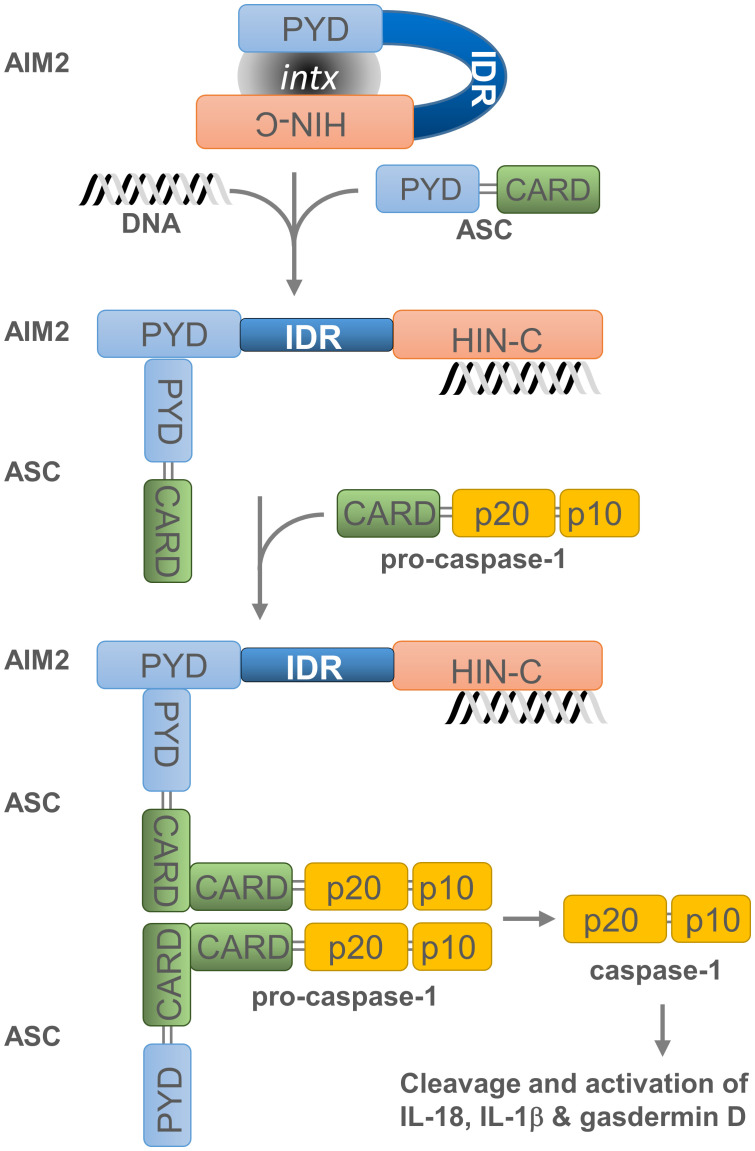
Assembly and activation of the AIM2 inflammasome. Overview of AIM2 inflammasome activation. Cytosolic dsDNA from invading pathogens or mislocated self-dsDNA (pathogen- or danger-associated molecular patterns: PAMPs or DAMPs) directly binds to the pattern recognition receptor (PRR) AIM2. This association, which relieves the intramolecular auto-inhibition imposed by the PYD-HIN-200 interaction, allows the recruitment of apoptosis-associated speck-like protein containing a CARD (ASC) as well as the binding and proximity-induced auto-proteolytic maturation of the pro-caspase-1. This inflammasome activation is likely to occur in the cytoplasmic compartment of the cell, where AIM2 is primarily located. Active caspase-1 can then cleave the pro-inflammatory cytokines IL-18 and IL-1β and the precursor of the pore-forming protein Gasdermin D (GSDMD). Cleaved GSDMD induces membrane pore formation, cell pyroptosis and the release of inflammatory cytokines. Grey oval indicated as ‘intx’ is a representation of the interaction between the PYD and HIN-200 (HIN-C) domains of AIM2.

PYHIN factors contain other structural components that are likely to influence their affinity for nucleic acids. The *in silico* analysis of MNDA (43) as well as IFI16, AIM2, and IFIX revealed that they all possess at least one putative intrinsically disordered region (IDR) located between their PYD and HIN-200 domain (**Figure 1**). Most IDRs can interact with DNA and RNA molecules (44, 45), and can therefore influence the nucleic acid binding properties of PYHIN factors. In addition, consecutive aspartate (D) or glutamate (E) residues (referred to as D/E repeats) within the IDR domains can change the conformation from an open to a closed state through intramolecular electrostatic interactions with other functional domains, thereby causing auto-inhibition (46). This mechanism of auto-inhibition is of particular interest for PYHIN proteins, since a putative IDR located between their PYD and HIN-200 domains could facilitate conformational changes that occur after nucleic acid binding to the HIN-200 domain, as has been reported for AIM2 (**Figure 2**) [(20, 46) and see below]. Such a conformational change could ‘free’ the PYD and facilitate its interaction with other PYD-containing proteins.

Like AIM2, IFI16 can directly target and bind viral genomic DNA or RNA (47–51). Furthermore, IFI16, IFIX/PYHIN1, and MNDA play an anti-viral role by sequestering or interfering with Sp1-dependent gene expression, which is required for the replication of the human immunodeficiency virus 1 (HIV-1), the hepatitis B virus, and the simian virus 40 (SV40) as well as retro-transposition of long interspersed element-1 (LINE-1) sequences (13, 52). Mechanistically, the PYD region of these factors has been reported to compete with DNA to bind the transcription factor Sp1, thereby displacing the latter factor from viral gene promoters and interfering with Sp1-dependent viral gene expression (13). Interestingly, MNDA is involved in the regulation of IFN-α expression (53), which could also explain why it can complement the effect of viral genome detection by cytosolic sensors (13, 53).

## Pattern recognition receptor activity of human PYHIN factors

AIM2 and IFI16 are the most thoroughly described PYHIN factors, and their biological importance has been well established. The characterization of their mechanisms of action has strongly influenced research into other PYHIN factors. The HIN-200 domains of AIM2 and IFI16 are essential for their interaction with pathogen dsDNA or dsRNA, and cytosolic mislocated self-DNA; because of this, the HIN-200 is critical for the activity of AIM2 and IFI16 as PRRs (29, 54), which is required to trigger inflammasome formation.

Untimely activation of AIM2-dependent inflammasome is prevented by several mechanisms. The auto-inhibition of AIM2 is due to the internal interaction between its PYD and HIN-200 domain (**Figure 2**). This inhibition is lifted when dsDNA binds to the HIN-200 domain which, in turn, enables dimerization of the AIM2 PYD with the PYD region of the inflammasome adaptor protein known as apoptosis-associated speck-like protein containing a CARD -or PYCARD- (ASC) (20). This PYD-PYD interaction favours the formation of a multimeric structure and permits the recruitment and activation of procaspase-1 through the CARD region of ASC. Subsequently, activated caspase-1 cleaves the pro-inflammatory cytokines interleukin 18 (IL-18) and 1β (IL-1β) (55–57) as well as the precursor of the pore-forming protein Gasdermin D (GSDMD) (58–61). This releases the N-terminal domain of GSDMD, which induces membrane pore formation, cell pyroptosis (or caspase-1-dependent and pro-inflammatory programmed cell death) (62), and the discharge of inflammatory cytokines (63).

While assembly and activation of the AIM2 inflammasome is dictated by the presence of cytosolic DNA, it is also dynamically influenced by interactions with other proteins. For example, PYD-only protein 3 (POP3) can inhibit AIM2 by interacting with its PYD region, thereby preventing the interaction of AIM2 with ASC and the consequent inflammasome formation (64). Similarly, the binding of murine Ifi202 to AIM2 interferes with AIM2-dependent signalling, preventing the assembly of the inflammasome that occurs after DNA recognition (65).

IFI16 has emerged as a component of an atypical, nuclear inflammasome. Upon detection of viral, microbial, or mislocated (cytoplasmic) self-DNA, IFI16 can form a functional AIM2-independent inflammasome that promotes caspase-1 activation and IL-1β cleavage (54). The PRR activity of IFI16 can also activate the stimulator of the interferon gene (STING) pathway in a cyclic GMP-AMP synthase (cGAS)-independent manner (30, 31, 66, 67). STING is associated with the endoplasmic reticulum (ER) and can detect cytosolic DNA or cyclic dinucleotides (68). Upon binding to its ligands, STING translocates from the ER to the Golgi (69, 70), where it recruits TANK binding kinase 1 (TBK1) also known as the nuclear factor kappa B (NF-κB) activating kinase. The TBK1 kinase activity elicits the nuclear relocalization and activation of NF-κB, as well as the phosphorylation, nuclear transfer and activation of interferon regulatory factor 3 (IRF3) (71–76). NF-κB then synergizes with IRF3 to activate the transcription of genes encoding type I IFNs and ISGs (77).

In addition to the above, a recent study showed that in response to viral RNA infection, IFI16 promotes retinoic acid-inducible gene l (*RIG1*) (also known as DEAD box protein 58 -*DDX58*-) gene expression and binds viral dsRNA through its HIN-A domain, which leads to a conformational change that facilitates the interaction between the PYD domains of IFI16 and RIG-1 (47). This interaction triggers RIG-1 polyubiquitination, activation, and association with mitochondrial antiviral signalling (MAVS) protein. This in turn triggers TBK1 activation, nuclear re-localization of IRF3, interferon regulatory factor 7 (IRF7), and NF-κB, and consequent transcriptional activation of ISGs and type I IFNs (53, 78, 79). Thoresen et al. (80) have shown that this RIG-1 signalling can occur rapidly during inflammation and is mechanistically distinct from RIG-1 oligomerization and aggregation that typically take place at the mitochondria membrane. The latter situation occurs subsequently, upon type I IFN induction, and leads to inflammasome activation as well as cell pyroptosis (62). Indeed, inflammasome activation and pyroptosis are usually observed at later stages of the acute phase of inflammation and during chronic inflammation [reviewed in (36, 81)].

The mechanism through which IFI16 coordinates inflammasome formation and the transcriptional activation of ISGs and type I IFN genes, is currently unclear. Interestingly, several IFI16 isoforms have been isolated (82). The short isoform IFI16-β, which does not include the PYD region, can interact with AIM2, thereby interfering with the formation of a functional AIM2-ASC complex (83). Thus, unlike the full-length protein, IFI16-β exerts a negative effect on inflammasome activation (54, 84, 85). IFI16-β contains both HIN-200 domains of the full-length protein and maintains the capacity to interact with and activate STING (83). Overall, the data suggests that IFI16-β synthesis during inflammation may dissociate the effect of IFI16 on ISG and type I IFN gene induction from its role in inflammasome activation.

Although IFIX and MNDA possess an HIN-200 domain, whether these proteins function as sensors of pathogen DNA is debated (23, 53, 86). It is also unknown whether they have the ability to interact with AIM2 and affect the AIM2 inflammasome activation, or if they can initiate inflammasome formation independently. Since IFIX and MNDA are located in the nucleus, they may contribute to the dynamics of the nuclear inflammasome or other aspects of the inflammatory process. Importantly, recent studies provide direct evidence that MNDA can control the expression of genes required for the cellular response to infection or sterile inflammation. This will be discussed more extensively below.

## Interactome and cellular localization of PYHIN factors

Proteomic and cellular localization studies have further defined the role of PYHIN factors. The protein interactome of individual overexpressed PYHIN factors in human HEK293 cells consists of over 300 proteins, with most interactors acting in intracellular signalling, cell cycle control, apoptosis, and transcriptional regulation. In addition, IFI16 and MNDA interactomes contain proteins involved in ribosome biogenesis and pre-rRNA processing, whereas IFIX and AIM2 strongly associate with proteins that modulate chromatin structure and DNA damage response (86). In the case of AIM2, these results are somewhat surprising: indeed, unlike IFI16 and IFIX, which contain a multipartite nuclear localization signal (NLS) (82, 87), or MNDA, which contains a putative NLS and shows a nuclear localization at steady state condition (88), AIM2 is mainly cytoplasmic. Nonetheless, AIM2 nuclear localization cannot be formally excluded (89), and the identified AIM2 interactome supports the idea that the cellular localization of PYHIN factors is relevant for their function.

Interestingly, while IFI16 and MNDA are predominantly nuclear, accumulation of these proteins in the cytosol has been detected in different cell types under certain conditions ( (31, 43, 90) and below). For instance, IFI16 has been detected in the cytoplasm of U2OS (91) and HeLa (92) cells, in UVB exposed keratinocytes (93), and in fibroblasts and endothelial cells upon infection with human herpesviruses, such as the Herpes simplex virus type 1 (HSV-1), the Kaposi’s sarcoma-associated herpesvirus (KSHV), and the Epstein-Barr virus (EBV) (54, 94–97). Once viral genomes are detected by IFI16 in the host cells, this PYHIN factor associates with the histone acetyltransferase p300, leading to the acetylation of several lysine residues within the IFI16 bipartite NLS (94, 98). These post-translational modifications impede the nuclear import of newly synthesized IFI16, thereby confining the protein to the cytoplasm.

MNDA is also relocated to the cytoplasm in response to signals related to the programmed cell death of neutrophils, and upon genotoxic stress or apoptosis induction in myeloid and lymphoid cells (43, 90). No post-translational modification has been associated with MNDA relocalization to the cytoplasm. Instead, cytoplasmic accumulation of MNDA has been linked to its cleavage by effector caspases (see following section).

## Cellular roles of MNDA and its relocalization during stress

MNDA has emerged as a stress-responsive factor whose localization undergoes dramatic changes during stress and following treatment with apoptosis-inducing agents. The predominant nucleolar localization of MNDA (43, 90, 99) was shown to be disrupted during programmed cell death and upon apoptosis induction or cellular stress induced by UVC radiation or cisplatin treatment (43, 90), with the protein being rapidly released into the nucleoplasm and subsequently relocated to the cytoplasm.

### Nuclear functions of MNDA

The ability of MNDA to bind dsDNA (100, 101) and its predominant localization at the nucleolus led authors to propose that it may act as a transcription factor (102). *In silico* analyses further suggested that it functions as a core transcription factor in monocytes (103, 104). Recent studies demonstrating that MNDA binds chromatin and modulates RNA polymerase II (Pol II) recruitment to myeloid cell leukaemia 1 (*MCL1*), B-cell lymphoma 2 (*BCL2*) and *IRF7* genes (43, 53) provided direct evidence that MNDA participates to transcriptional regulation in leukocytes. While the HIN-200 domain and/or IDR are expected to be required for genomic DNA binding, no putative DNA binding site or consensus DNA sequence has been identified for MNDA. The assistance of other interacting partners, such as *bona fide* transcription factors (e.g., Yin Yang 1, or YY1) (105), may therefore be required for the targeting of MNDA to specific genes.

A critical step in the release of promoter-paused Pol II is the phosphorylation of negative elongation factor (NELF) and of the C-terminal domain (CTD) of Pol II by cyclin dependent kinase 9 (CDK9). This kinase is a component of the positive transcription elongation factor b (P-TEFb) complex, a key regulator of pre-mRNA synthesis of most genes in mammals (106–110). Bottardi et al. demonstrated that MNDA interacts with CDK9 and hexamethylene bis-acetamide-inducible protein 1 (HEXIM1) in myeloid and lymphoid cells, where it acts to control the expression of *MCL1* and *BCL2* genes (43). Indeed, in response to chemotherapeutic agents that induce genotoxic stress, MNDA binding to *MCL1* and *BCL2* pre-mRNAs is associated with lower levels of transcripts and consequently, MCL-1 and BCL-2 proteins. The effect of MNDA at these genes is not linked to variations in chromatin structure or to the compromised formation of the transcriptional pre-initiation complex. Instead, MNDA impairs optimal recruitment and distribution of Pol II and P-TEFb at the transcription start site and open reading frame of these genes. The peculiar chromatin distribution of Pol II and P-TEFb observed at specific genes in MNDA expressing cells (43), strongly suggests that MNDA hampers efficient transcriptional elongation by reducing Pol II processivity and affecting the reloading of P-TEFb and Pol II (43, 111). Overall, MNDA-dependent regulation of Pol II productive elongation and processing of RNA molecules contribute to fine-tuning the transcription of specific genes in both normal and stress-induced conditions.

### Cytoplasmic functions of MNDA

**Figure 3:**
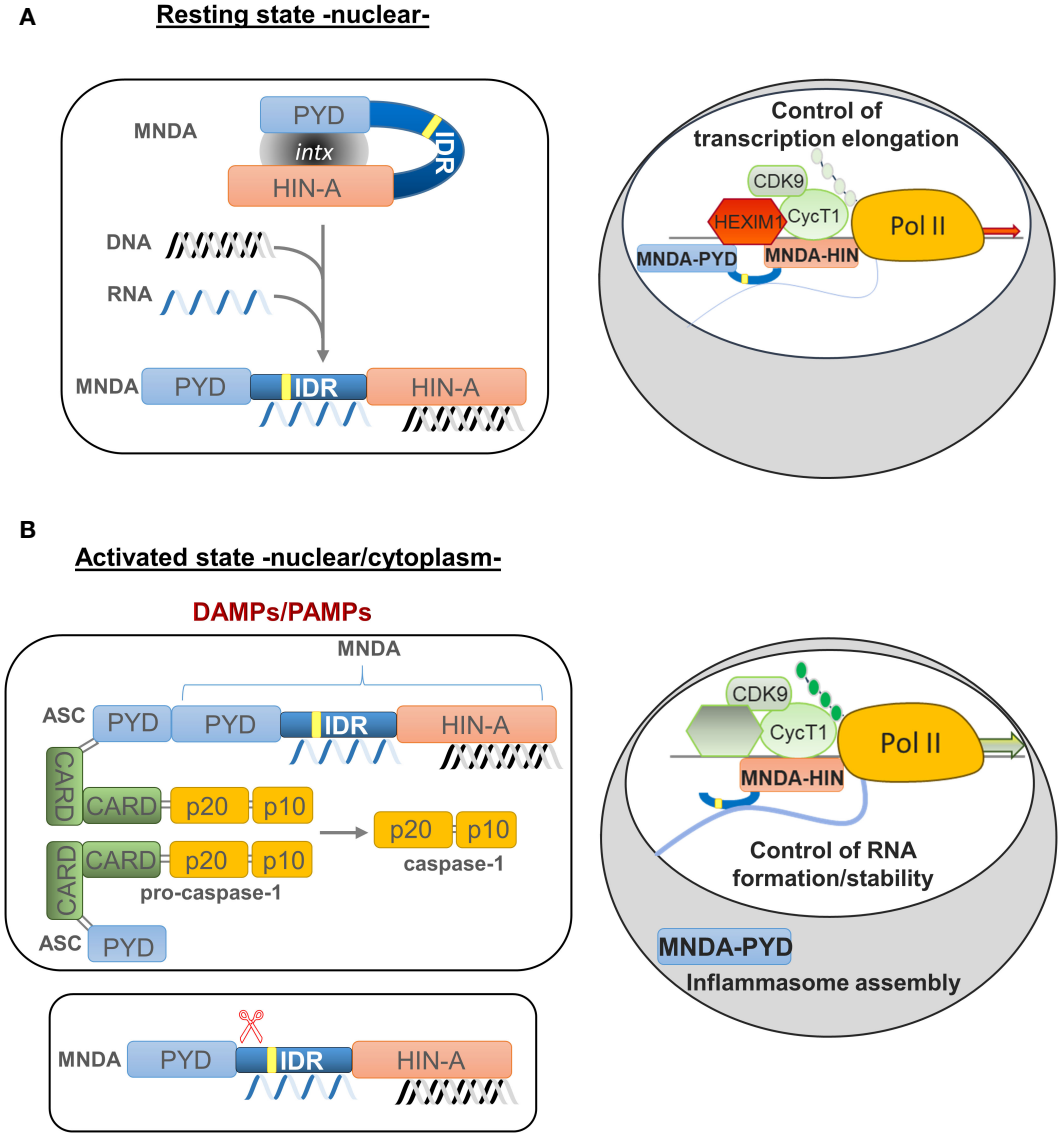
Model of MNDA nuclear function, cleavage and cytoplasmic relocalization. MNDA’s cellular localization and function vary in response to endogenous and exogenous stress signals. **(A)** Under normal conditions (resting state), MNDA’s PYD and HIN-200 (HIN-A) domains form an intramolecular association that results in a self-stabilized “resting” state, with the flexible link of the intrinsically disordered region (IDR) buckling. DNA binding to the HIN-200 domain and/or RNA association with the IDR or HIN-200 domain in the nucleoplasm weaken this intramolecular interaction and release constraints on the PYD signalling domain. The latter can then engage in homotypic or non-homotypic PYD-PYD interactions, for example with the nuclear protein ASC (apoptosis-associated speck-like protein containing a CARD) (not shown). MNDA can also associate with various nuclear proteins (86), including activating or repressive components of the positive transcription elongation factor b (P-TEFb) complex (43). In this “resting” state, MNDA could restrict the release of the promoter-proximal paused RNA polymerase II (Pol II) and, consequently, negatively affect transcriptional elongation of these genes. Grey oval indicated as ‘intx’ is a representation of the interaction between the PYD and HIN-200 (HIN-A) domains of MNDA. **(B)** In response to genotoxic or inflammation mediators such as danger- or pathogen-associated molecular patterns (DAMPs or PAMPs) (activated state), PYD-PYD interactions between MNDA and ASC proteins become possible, triggering inflammasome formation and, consequently, activation of pro-caspase-1. Among other substrates, catalytically active caspase-1 might induce proteolytic cleavage of MNDA. This would generate at least two characteristic MNDA fragments (43, 90). While the PYD-only fragment relocalizes to the cytoplasm where it may facilitate inflammasome assembly, the ‘IDR-HIN’ fragment would accumulate in the nucleus. Although it is not known whether the absence of PYD weakens the interaction of MNDA-HIN with repressive P-TEFb components such as HEXIM1, it has been shown that MNDA-dependent repression of transcription elongation is relieved following genotoxic stress (43). At the same time, MNDA may help stabilize newly transcribed RNA. This dual role of MNDA and the effect of MNDA on transcription elongation of MNDA targets, such as *MCL1* and *BCL2*, could therefore be modulated by signals induced by genotoxic and inflammatory mediators.

Caspase-dependent cleavage of MNDA is associated with its relocalization and accumulation in the cytoplasm (90). The size of the MNDA protein fragments formed during caspase cleavage suggests that the PYD and HIN-200 domains are then separated into two different protein fragments (43, 90) (**Figure 3**). If, as in the case of AIM2, the PYD region of MNDA interacts with and is encumbered by its HIN-200 domain, this caspase-dependent cleavage may release the PYD region of MNDA, and promote its interaction with the PYD of ASC, which is required for formation and activation of the inflammasome (37) (**Figures 2**, **3**). Further experiments will be necessary to define the precise effect of this cleavage and to identify the mechanism regulating this cleavage and, therefore, MNDA proteostasis.

The relocalization of MNDA from the nucleus to the cytoplasm correlates with the induction of effector caspases and cell death in circulating human neutrophils (90). Moreover, prolonged neutrophil survival, which results in the uncontrolled release of pro-inflammatory molecules and can lead to neutrophil-induced severe tissue injury propagating sepsis and possibly inducing septic shock (112–115), prevents MNDA cleavage and its cytoplasmic accumulation (90). Conversely, neutrophil cell death correlates with MNDA cleavage and accumulation in the cytoplasm. Likewise, in the myeloid cell line HL-60 exposed to genotoxic agents, MNDA expression promotes proteasomal degradation of the anti-apoptotic factor MCL-1 and favours apoptosis (90, 116). In agreement with these observations, MNDA also controls MCL-1 and BCL-2 protein and transcript levels in normal and leukemic B-cells, whereby higher MNDA expression levels are associated with markers of good clinical outcome (16, 43).

## MNDA deregulation and diseases

**Figure 4:**
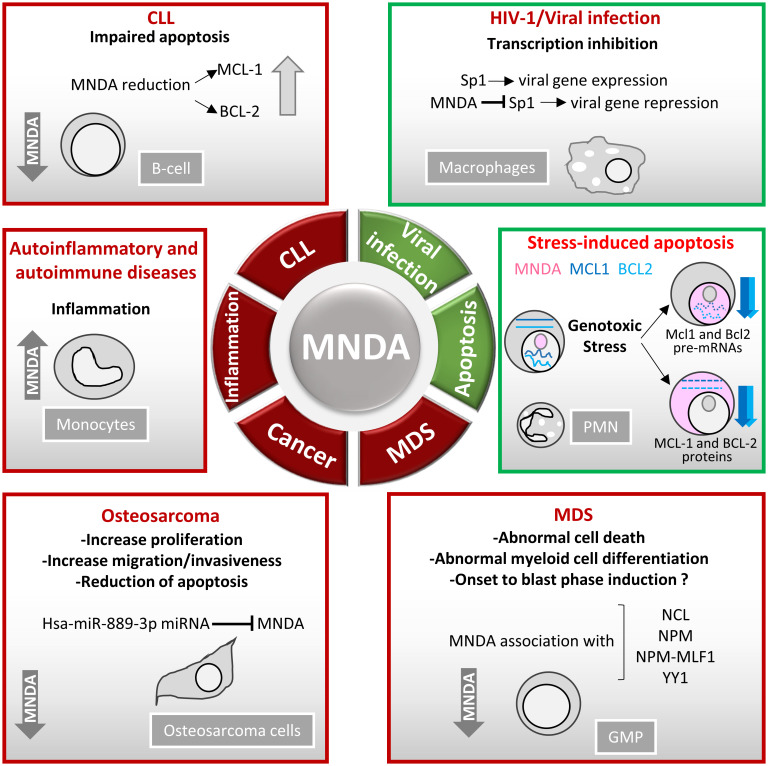
MNDA expression levels in human diseases. Schematic overview of the role of MNDA expression in human diseases and cellular functions. **-**In macrophages, MNDA can inhibit the DNA binding of the transcription factor Sp1 and thereby, blocks virus replication during human immunodeficiency virus 1 (HIV-1) infection (13). **-**Upon genotoxic stress in chronic lymphocytic leukaemia (CLL) cells or polymorphonuclear (PMN) leukocytes, MNDA represses *MCL1* and *BCL2* gene expression and enhances MCL-1 and BCL-2 protein degradation, promoting stress-induced apoptosis (90). -In granulocyte-monocyte progenitor (GMP) cells, reduced MNDA protein levels is associated with abnormal cell death and myeloid differentiation, and has been documented in familial (133) and sporadic (132–135) myelodysplastic syndromes (MDS). MNDA binds nucleophosmin 1 (NPM1) and the NPM-MLF1 (myeloid leukaemia factor 1) chimera product (99), which has been associated with MDS (136). Whether these associations alter trafficking signals and/or direct inappropriate cellular localization of the aforementioned proteins in MDS cells remains to be elucidated. MNDA also interacts with nucleolin (NCL) and Yin Yang 1 (YY1) proteins, which are involved in cell cycle regulation and genotoxic stress response (137, 138), suggesting a possible role of MNDA in the control of MDS cell survival. In accordance with this data and regardless the fact that the precise mechanism of action is still undefined, MNDA expression was proposed as an independent clinical marker for the evaluation of dyspoiesis (139). -In osteosarcoma cells, the Hsa-miR-889-3p microRNA, whose upregulation is a known prognostic risk factor (130), inhibits MNDA expression whereas MNDA overexpression counteracts proliferation, induces apoptosis and reduces migration and invasiveness in the SAOS-2 osteosarcoma model cell line (131). -MNDA overexpression has been documented in monocytes obtained from individuals diagnosed with autoinflammatory (atherosclerotic lesions) (126); autoimmune (pancreatic islets of Langerhans in type 1 diabetes) (129); chronic inflammatory diseases such as ulcerative colitis (127) and chronic obstructive pulmonary disease (128).

Consistent with their role as DNA sensors and immune activators, PYHIN factors are essential for rapid and powerful responses against endogenous and exogenous threats. Moreover, these factors must be tightly regulated to avoid inadequate innate immune response and dysregulated inflammasome activation. Accordingly, various genetic deletions of the human chromosomal 1q21-23 region, where *MNDA* and other PYHIN genes are clustered, have been linked to the development of diseases including breast and prostate cancers (21). Furthermore, the integrity of this chromosomal region is thought to be relevant to the regulation of acute and chronic inflammatory responses as well as autoimmune susceptibility [(117) and references therein]. Beside these large-scale rearrangements, point mutations and copy number variation of the *MNDA* gene have been identified in various hematopoietic cancers (**Table 1**). The association of abnormal MNDA expression levels and the onset of hematopoietic and inflammatory disorders are also consistent with the notion that MNDA dosage is critical during haematopoiesis (**Table 2**; **Figure 4**).

**Table 1:** MNDA mutations and expression level variations in hematopoietic malignant neoplasms.

A					
		Frequency (%)	Samples tested	Type of cancer	References
Point mutations		0.40%	7278	Section B	Section B
Copy number variations		0.15%	661	Diffuse large B-cell lymphoma	(118)
Gene expression		4.07%	221	Diffuse large B-cell lymphoma Acute myeloid leukaemia	(118)
B					
**AA**	**Domain of MNDA**		**Type of Cancer**		**References**
V59I	PYD		Acute lymphoblastic B-cell leukaemia		(119)
A112E	IDR		Acute myeloid leukaemia		–
T178I	IDR		Plasma cell myeloma		(120)
P183L	IDR		Plasma cell myeloma		(120)
P190L	IDR		Plasma cell myeloma		(120)
V214M	HIN A		T-cell prolymphocytic leukaemia		(121)
V215M	HIN A		Acute myeloid leukaemia		–
P296T	HIN A		Hairy cell leukaemia		(122)
L367V	HIN A		Chronic lymphocytic leukaemia-small lymphocytic lymphoma		(123)
P403L	HIN A		T-cell prolymphocytic leukaemia		(121)
NS			NK-T cell lymphoma		–
NS			Diffuse large B-cell lymphoma		–
NS			Mycosis fungoides-Sezary syndrome		(124)
NS			Lymphoid neoplasm		(118)
NS			T-cell large granular lymphocytic leukaemia		(125)

**A:** Genetic alterations and expression variations of the MNDA protein found in haematopoietic cancers (COSMIC Database; https://cancer.sanger.ac.uk/cosmic; update February 2024).

**B:** Somatic mutations in MNDA protein; 41.38% of the mutations correspond to missense substitutions. The amino acid (AA) position substitution are specified. The MNDA domain affected by the mutations is indicated; NS, not specified.

**Table 2:** MNDA expression levels and functions in various human diseases.

Disease	Role of MNDA	Reference
Autoinflammatory, chronic inflammatory, and autoimmune diseases	• MNDA is overexpressed in monocytes at sites of autoinflammatory (atherosclerotic lesions) (126), chronic inflammatory (ulcerative colitis (127); chronic obstructive pulmonary disease (128) and autoimmune (pancreatic islets of Langerhans in type I diabetes) (129) diseases.	(126–129)
Osteosarcoma	• The prognostic risk factor Hsa-miR-889-3p inhibits MNDA expression (130). MNDA inhibits proliferation, induces apoptosis and reduces migration/invasiveness of Saos-2 osteosarcoma cells (131).	(130, 131)
Chronic lymphocytic leukaemia	• MNDA decreases the expression levels of BCL-2 and MCL-1, and decreases B-cell survival.	(43)
HIV-1	• MNDA expression restricts retroviral gene expression by sequestering the transcription factor Sp1 in human macrophages and CD4^+^ T-cells.	(13)
Myelodysplastic syndrome	• Downregulation of MNDA expression is associated with excessive programmed cell death in myeloid progenitors, which results in a decrease of mature effector cells.	(132)
COVID-19	• MNDA expression correlates with the inflammatory levels of critically ill COVID-19 patients (no dexamethasone treatment).	(28)
Sepsis and septic shock	• MNDA expression triggers the degradation of MCL-1 and favors neutrophil apoptosis	(90)
Non-Hodgkin lymphomas	• MNDA can serve as diagnostic tool for the differential diagnosis of non-Hodgkin lymphoma subtypes. MNDA is highly expressed in nodal marginal zone lymphoma (NMZL) but not in follicular lymphoma (FL). MNDA function in these pathologies is still unknown.	(17)

### Inflammation and cancer

An association between PYHIN dysregulation and inflammatory disorders, e.g., autoimmune diseases and cancers, has been proposed to reflect PYHIN propensity to act as PRRs that sense and bind both exogenous and mislocated self-DNA (29, 140). Although there is no evidence that MNDA can act as a classical PRR (53), the regulation of transcription and apoptosis by this protein is likely to be important to control the inflammation response to infection and host cell damages. This view is supported by the strong correlation between the expression levels of MNDA and inflammation-related diseases and cancers. Indeed, MNDA is overexpressed in peripheral blood mononuclear cells of patients diagnosed with autoinflammatory diseases, such as inflammatory spondyloarthropathy (141); autoimmune diseases, like rheumatoid arthritis and psoriatic arthritis (141); and Sjögren patients (10, 142, 143). In line with the evidence that MNDA is overexpressed in macrophages at sites of inflammation (144), the number of MNDA positive monocytes in atherosclerotic aorta has been shown to increase during disease progression (126). The expression of MNDA is also significantly higher at inflammation sites in pancreatic islets of Langerhans isolated from type 1 diabetes patients, especially those developing complications like neuropathy [reviewed in (145)].

Cancer can be regarded as an inflammation-related disease. In particular, solid tumours are surrounded by stromal and inflammatory cells, which form the tumour microenvironment. In ‘hot’ tumours, this pro-inflammatory milieu can restraint tumour growth and enhance immunotherapeutic efficiency. However, in ‘cold’ tumours (146), the predominant myeloid-related innate immunity signature leads to the production of different mediators that can increase genomic DNA damage, and render the tumour cells refractory to immune checkpoint inhibitors and DNA repair mechanisms (147–149) while favouring immunotherapeutic resistance. It is therefore possible that factors modulating inflammation and hematopoietic cell death, such as MNDA, may act to limit cancer progression by controlling the extent of inflammation.

Mechanistically, cancer biology and inflammation rely on trained immunity, which is defined as the acquisition of memory by innate immune cells. It is a functional state that is associated with potent immune responses and can elicit robust adaptive immunity against infection and cancer. Initially considered to rely on monocytes and macrophages, trained immunity is also documented in neutrophils, natural killer (NK) cells, innate lymphoid cells and non-immune cells, like hematopoietic precursors, endothelial and epithelial cells (150). PYHIN proteins are expressed in epithelial cells (151, 152), which are nowadays considered as trained immunity-associated innate immune cells (153). Their possible roles as initial sensing of PAMPs and DAMPs and induction of ISG transcription in airway epithelial cells has been recently highlighted, in particular during respiratory viral infection such as caused by the single-stranded positive-sense RNA viruses known as severe acute respiratory syndrome coronavirus 2 (SARS‑CoV‑2) (154). Entry of SARS-CoV-2 into epithelial cells impairs the RIG-1 and melanoma differentiation-associated gene 5 (MDA-5) signalling and the localized first peak of IFN response. As a result, the required antiviral environment is not established and the ISG/IFN response, if any, must be assured by the underneath sentinel immune cells, such as macrophages. If these cells are not functionally intact and carry instead genetic defects (as described in young men with dysfunctional TLR7-variants), the IFN response by the host can be inadequate and dangerous [reviewed in (154)]. The specific example of SARS-CoV-2 infection emphasizes the role of PYHIN in cells others than sentinel immune cells, where they can add an extra layer of immunoprotection somehow specific to the tissue and type of perturbation. It is worth noting that it has been reported that the MNDA expression levels correlate with increased inflammatory levels during the acute phase of the disease (28).

### Myelodysplastic syndromes

The elevated expression of MNDA is a marker of various forms of myelodysplastic syndromes (MDS), which comprise erythro-myelopoiesis disorders. Indeed, MNDA expression has been proposed as an independent clinical marker for the evaluation of myeloid dyspoiesis (139). MDS are characterized by a loss of responsiveness of hematopoietic progenitor cells to environmental signals, as well as excessive apoptosis and/or susceptibility to programmed cell death (PCD) (155). PCD can be further classified as apoptotic or non-apoptotic cell death. The latter can take various forms, including pyroptosis, which is an immunoreactive form of cell death following recognition of intracellular pathogen sensors such as AIM2 or the nucleotide oligomerization domain (NOD)-like receptor (NLR) sensors. Unlike apoptosis, which is acknowledged as a form of non-inflammatory PCD, pyroptosis is mainly regulated by inflammation-related caspases, including caspase-1 that, upon activation, mediates membrane pore formation through the cleavage of GSDMD (see above) [reviewed in (156)]. It has been reported that a hallmark of MDS is the assembly of the NOD-like receptor protein 3 (NLRP3) inflammasome and the pyroptotic death of hematopoietic stem and progenitor cells, which can favour the cytopenia that marks the first phase of the disease (157). This is followed by a second or blastic phase, characterized by the accumulation of myeloid progenitors and reduced cell death (158). In approximately one third of the cases, MDS progresses to acute myeloid leukaemia (AML).

Granulocyte-macrophage progenitor cells isolated from familial and sporadic MDS patients present reduced MNDA expression (133–135). MDS cells are particularly sensitive to tumour necrosis factor (TNF)-related apoptosis inducing ligand (TRAIL)- apoptosis, a phenomenon that can be prevented in the K562 cell model by ectopic expression of MNDA (132). The effect of MNDA is probably specific to TRAIL-induced apoptosis during MDS since MNDA cannot protect K562 cells from caspase-dependent apoptotic inducers, *e.g*., H_2_O_2_ (132). In accordance with this, MNDA was demonstrated to act as a pro-apoptotic factor in several myeloid and lymphoid cell lines (90, 159).

### Leukaemia and lymphoma

Treatment regimens differ significantly based on the type of haematological disorder. Therefore, it is important to identify disease-specific biomarkers and characterize the molecular mechanisms behind these pathologies. Despite significant progress in biomarker classification (160, 161), several haematological diseases still lack specific biomarker profiles. For instance, the spreading of AML cells to different tissues and organs, which is referred to as extramedullary (EM) involvement (162), can be misdiagnosed and confused with large cell lymphoma or other lymphoproliferative disorders. In addition, EM AML are morphologically similar to blastic plasmacytoid dendritic cell neoplasm (BPDCN), which also arises at EM sites (163). Interestingly, MNDA can be detected by immunohistochemistry in the majority of EM AML tested, whereas in all cases of BPDCN, MNDA expression is undetectable (164).

MNDA can also be used as a diagnostic tool to distinguish between various forms of indolent low-grade lymphomas, which regroup slow growing mature B-cell non-Hodgkin lymphomas (NHL) such as marginal zone lymphoma (MZL) and follicular lymphoma (FL). MNDA is highly expressed in MZLs (nodal, extranodal, splenic) and in normal primary follicles (165), but is uncommon in FL (17, 166, 167).

Chronic lymphocytic leukaemia (CLL)/small lymphocytic lymphoma (SLL), which is a type of NHL, is a malignancy of monoclonal, mature, antigen-experienced, and apoptosis refractory CD19^+^/CD5^+^ B lymphoid cells, detected in peripheral blood at a concentration of ≥5000 cells/μL for a period of 3 months or more (168). This disease is called CLL when most of cancer cells are in the blood and bone marrow and SLL when they localize to the lymph nodes and spleen. However, these terminologies represent different forms of the same illness (which will be referred to as CLL hereafter). CLL is characterized by a heterogeneous clinical course. Numerous investigations indicate that CLL relapse is commonly associated with defects in pathways regulating the DNA damage response (in particular, mutations or deletion of the p53 tumour suppressor), inhibition of apoptosis (commonly caused by the overexpression of the BCL-2 family of anti-apoptotic proteins), and RNA metabolism (often affecting the splicing factor 3b subunit 3 -SF3B3-). Molecular and cytological defects have been regarded as direct consequences of the accumulation of somatic gene mutations acquired over time (169–171), as well as being the result of global and cumulative changes in the epigenetic landscape (172). Further characterization of known CLL biomarkers (173–176) and the identification of new biomarkers would be expected to improve prognostic accuracy, staging, and setting of novel therapeutic avenues. Unfortunately, with the notable exception of chromosome 13 focal deletion [del(13q)] (177), the functional impact of the somatic events that progressively transform normal B-cells towards a leukemic state have only been partially characterized, and because of this are by themselves inadequate to accurately define the disease outcome. Interestingly, the expression level of MNDA has been proposed as a predictive marker of the clinical progression of CLL (16, 43, 178). At the molecular level, an inverse correlation between the expression levels of MNDA and that of the anti-apoptotic proteins MCL-1 and BCL-2 has been observed in normal B- and CLL cells, confirming the importance of MNDA in the transcriptional control of *BCL2* and *MCL1* genes (43). CLL clones isolated from patients exhibiting reduced life expectancy and/or shorter disease-free period are indeed characterized by lower MNDA expression levels (16, 43). This observation is noteworthy since BCL-2 overexpression is found in most B-cell lymphoproliferative disorders, including CLL, and is responsible for the ‘apoptotic block’, which prevents cancer cells from initiating programmed cell death (179, 180). To counteract BCL-2 overexpression, BH3 mimetic drugs (like Venetoclax) and next generation BCL-2 inhibitors are suitable therapeutic choices (181, 182). However, their use in prolonged treatment frequently leads to resistance and clinical relapse. Among the best characterized mechanisms of resistance, are acquired BCL-2 mutations that reduce Venetoclax binding to BCL-2 (183), and overexpression of MCL-1 and BCL-XL proteins (184–186). Accordingly, MCL-1 overexpression is acknowledged as a negative prognostic marker in CLL and AML (43, 187–189). To counteract MCL-1 overexpression in refractory CLL, several MCL-1 inhibitors have been tested (190), either alone (191) or in combination with Venetoclax (192). However, MCL-1 inhibitors have been associated with significant side effects including cardiotoxicity (193, 194), limiting their therapeutic suitability. *MCL1* transcriptional block has been assessed as clinical alternative, but this approach affects transcription broadly and cause toxic side effects [(149, 195) and references therein]. The finding that, in CLL cells, high MNDA expression is associated with downregulation of the anti-apoptotic factors MCL-1 and BCL-2 and with improved clinical course is promising. Further investigation is therefore justified to decipher how MNDA expression is controlled in CLL clones, and to fully define the molecular contribution of MNDA to the outcome of this disease.

## Conclusions and open questions

In leukocytes, a precise coordination between apoptosis control and inflammation is required to regulate the response to PAMPs and DAMPs. The latter are primarily produced upon cell death resulting from infections or trauma, such as those caused by tissue lesions or genotoxins. DNA damage can be engendered by pathogens such as bacteria (reviewed in (196)), environmental pollution (like silica and asbestos particles, polycyclic aromatic hydrocarbons, pesticides, ionizing radiations, UV light, *etc.*), and anti-cancer drugs (such as alkylating agents) [(197) and references therein]. PAMPs and DAMPs trigger intracellular signalling leading to the activation of IFN genes and ISGs. IFN-regulated factors, including the PYHIN proteins, are therefore instrumental for innate and adaptive immune response to pathogen infection and also, to coordinate sterile inflammation (198). MNDA appears unlikely to act as a cytoplasmic DNA sensor, and does not activate the STING pathway or the ASC inflammasome cascade in HEK293 or THP-1 cells (53, 199). As discussed previously, the most important role of MNDA during inflammation is probably related to transcriptional control of genes required for the timely regulation of mechanisms governing the cellular response to infection or tissue damage. MNDA is not the unique PYHIN whose primary importance would be related to transcriptional regulation instead of PRR activity. Indeed, the mouse PYHIN protein Ifi207 is not involved in DNA sensing, but its association with active RNA polymerase II (RNA Pol II) and IRF7 in the nucleus is reported to enhance the transcriptional activation of IRF7-dependent cytokines, including the TNF gene in macrophages (200).

MNDA can induce *IRF7* transcription, while repressing *MCL1* and *BCL2* transcription. The effect of MNDA on *IRF7* results in increased expression of the type I interferon IFN-α (53). In addition to their critical role during inflammation, type I IFNs can synergize with apoptosis-promoting factors to increase caspases expression and create a molecular environment favourable for apoptosis (201). Thus, along with down-regulation of prosurvival *MCL1* and *BCL2* genes, *IRF7* transcriptional induction may also be required for MNDA-mediated regulation of apoptosis in hematopoietic cells. Another important finding is that cytoplasmic MNDA favours proteasomal degradation of MCL-1, thereby promoting apoptosis (90). Interestingly, the beneficial proapoptotic effect of MNDA has been demonstrated in leukocytes during sepsis and in CLL cells, two haematological disorders characterized by dysregulated apoptosis.

Several important questions concerning the biology of MNDA and other PYHIN factors remain unanswered. Among them, their transcriptional regulation and the consequence of specific signalling events on PYHIN synthesis, stability and functional activation deserve further scrutiny. Investigation aimed at deciphering the intramolecular interactions between the PYD and HIN-200 domain, as well as the interactions between the various PYHIN factors, are expected to provide valuable information regarding the regulation of this family of factors. Further work is needed to explore how post-translational modifications can modulate the proteome of PYHIN factors and influence their cellular localization and function. Finally, since MNDA responds to different types of stress and controls the transcription of *IRF7*, it will be important to define whether this protein can influence cellular functions other than apoptosis, and to elucidate its importance in the context of innate and adaptive immune responses.

Whether acting as a PRR or stress-responsive transcriptional regulator, MNDA, together with the other PYHIN factors, are required for a timely response to pathogen infection or sterile inflammation. Further characterization of PYHIN factors is expected to lead to the development of novel therapies for haematological disorders characterized by abnormal apoptosis and inflammatory response.

## Author contributions

SB: Conceptualization, Writing – original draft, Writing – review & editing, Visualization. TL: Writing – original draft, Writing – review & editing. AR: Writing – review & editing, Visualization. NQ: Visualization, Writing – review & editing. HW: Writing – review & editing, Funding acquisition. EA: Funding acquisition, Writing – review & editing. EM: Conceptualization, Funding acquisition, Writing – original draft, Writing – review & editing.

## Glossary

**Table:**

**AML**	Acute myeloid leukaemia is a cancer of the myeloid line of blood cells that is characterized by clonal expansion of immature blast cells in the peripheral blood and bone marrow resulting in ineffective erythropoiesis and bone marrow failure.
**Autoinflammatory diseases**	They are a group of disorders caused by a dysregulation of the innate immune system leading to episodes of systemic inflammation.
**Autoimmune diseases**	They are conditions that result from malfunction of the adaptive immune system. It is estimated that there are more than 80 recognized autoimmune diseases, with recent scientific evidence suggesting the existence of potentially more than 100 distinct conditions.
**Blastic phase**	Blastic phase is a leukemic transformation that can occur in myeloproliferative neoplasms (MPN). Also referred to as “blast-phase MPN”.
**Blastic plasmacytoid dendritic cell neoplasm**	Blastic plasmacytoid dendritic cell neoplasm (BPDCN) is a highly aggressive malignancy derived from the precursors of plasmacytoid dendritic cells, immune cells that specialize in the production of type I interferons in response to bacterial and viral stimuli.
**CLL/SLL**	Chronic lymphocytic leukaemia (CLL) and small lymphocytic lymphoma (SLL) are cancers that affect lymphocytes. When most of the cancer cells are located in the bloodstream and the bone marrow, the disease is referred to as CLL, although the lymph nodes and spleen are often involved. When the cancer cells are located mostly in the lymph nodes, the disease is called SLL.
**Cytopenia**	Cytopenia is a reduction in the number of mature blood cells in the blood.
**Extramedullary involvement**	Extramedullary involvement (EMI) refers to leukemic cells found in organs or tissue outside the blood or bone marrow. The most common sites of extramedullary disease are skin, bone, and lymph nodes.
**Follicular lymphoma**	Follicular lymphoma (FL) is typically a slow-growing or indolent form of non- Hodgkin lymphoma (NHL) that arises from B-lymphocytes, making it a B-cell lymphoma. This lymphoma subtype accounts for 20 to 30 percent of all NHL cases and is categorized as more of a chronic disease.
**Inflammatory spondyloarthropathy**	Spondyloarthritis is a family of inflammatory rheumatic diseases characterized by inflammation in the spine (“spondylitis”) and joints (“arthritis”).
**Marginal zone lymphoma**	Marginal zone lymphoma (MZL) is a group of indolent non-Hodgkin B-cell lymphomas.
**Myelodysplastic syndromes (MDS)**	MDS are a group of heterogeneous myeloid disorders of the bone marrow defined by the presence of disordered cellular maturation (**myeloid dyspoiesis**), with clinical, morphological, and genetic features that are shared by other myeloid disorders. MDS can progress from the chronic phase to an accelerated blastic phase.
**Non-Hodgkin lymphoma**	Non-Hodgkin lymphoma (NHL) is a type of cancer that originates from lymphocytes and generally develops in the lymph nodes and lymphatic tissue found in organs such as the stomach, intestines or skin. In some cases, NHL involves bone marrow and blood. NHL has many different subtypes which are either indolent (slow-growing) or aggressive (fast-growing).
**Psoriatic arthritis**	Psoriatic arthritis is one type of spondyloarthritis, linked with psoriasis, a chronic skin and nail disease.
**Rheumatoid arthritis**	Rheumatoid arthritis is a common, autoimmune disease that causes inflammation and pain in multiple joints of the body.
**Sjögren’s syndrome**	Sjögren’s syndrome is an autoimmune disease that mainly targets moisture-producing glands.
**Systemic lupus erythematosus**	Systemic lupus erythematosus is a chronic autoimmune disease that can affect many parts of the body (skin, joints, heart, lung, kidneys, circulating blood cells, and brain).
**Systemic sclerosis**	Systemic sclerosis or scleroderma is an autoimmune disease that causes inflammation and fibrosis. Localized scleroderma only affects the skin and the structures directly under the skin. Systemic sclerosis, also called systemic scleroderma, affects many systems in the body, such as the heart, lungs, and kidneys.
**Ulcerative colitis**	Ulcerative colitis is a chronic inflammatory bowel disease in which abnormal reactions of the immune system cause inflammation and ulcers on the inner lining of the large intestine.
[del(13q)]	chromosome 13 focal deletion
AIM2	absent in melanoma 2
ALRs	AIM2-like receptors
AML	acute myeloid leukaemia
ASC	apoptosis-associated speck-like protein containing a CARD or PYCARD
B-cell	lymphoid B cell
BCL2 and BCL-2	B-cell lymphoma 2 gene and protein
BPDCN	blastic plasmacytoid dendritic cell neoplasm
CDK9	cyclin dependent kinase 9
cGAS	cyclic GMP-AMP synthase
CLL	chronic lymphocytic leukaemia
CTD	C-terminal domain
D/E repeats	consecutive aspartate (D) or glutamate (E) residues
DAMPs	damage-associated molecular patterns
DDX58	DEAD box protein 58
ds	double-stranded
EBV	Epstein-Barr virus
EM	extramedullary
FL	follicular lymphoma
GMP	granulocyte-monocyte progenitor
GSDMD	gasdermin D
HEXIM1	hexamethylene bis-acetamide-inducible protein 1
HIV-1	human immunodeficiency virus 1
HSV-1	herpes simplex virus type 1
IDR	intrinsically disordered region
IFI16	IFN-γ inducible protein 16
Ifi-200/HIN-200	IFN inducible p200/hematopoietic IFN-inducible nuclear protein with a 200-amino-acid repeat domain
IFIX/PYHIN1	IFN-inducible protein X/pyrin and HIN domain-containing protein 1
IFN	type I and type II interferon
IL-1b	interleukin-1b
IL-18	interleukin-18
IRF3	interferon regulatory factor 3
IRF7	interferon regulatory factor 7
ISGs	IFN-stimulated genes
KSHV	Kaposi’s sarcoma-associated herpesvirus
MCL1 and MCL-1	myeloid cell leukaemia 1 gene and protein
MDA	melanoma differentiation-associated gene 5
MDS	myelodysplastic syndrome
MLF1	myeloid leukaemia factor 1
MNDA	myeloid nuclear differentiation antigen
MZL	marginal zone lymphoma
NCL	nucleolin
NELF	negative elongation factor
NF-kB	nuclear factor kappa B
NHL	non-Hodgkin lymphomas
NK	natural killer
NLRP3	NOD-like receptor protein 3
NOD	nucleotide oligomerization domain
NPM1	nucleophosmin 1
OB	oligonucleotide/oligosaccharide binding
PAMPs	pathogen-associated molecular patterns
PMN	polymorphonuclear
Pol II	RNA polymerase II
POP3	PYD-only protein 3
PRR	pattern-recognition receptor
P-TEFb	positive transcription elongation factor b
PYD/DAPIN	PYRIN domain/ domain in apoptosis and interferon response
PYHIN	PYRIN and HIN-200 domains
RIG-I	retinoic acid-inducible gene I
SF3B3	splicing factor 3b subunit 3
SLL	small lymphocytic lymphoma
ss	single-stranded
STING	stimulator of the interferon gene
SV40	simian virus 40
TBK1	TANK binding kinase 1
TRAIL	TNF-related apoptosis inducing ligand
TNF	tumour necrosis factor
YY1	Yin Yang 1
